# Co-complex protein membership evaluation using Maximum Entropy on GO ontology and InterPro annotation

**DOI:** 10.1093/bioinformatics/btx803

**Published:** 2018-01-30

**Authors:** Irina M Armean, Kathryn S Lilley, Matthew W B Trotter, Nicholas C V Pilkington, Sean B Holden

**Affiliations:** 1 Department of Biochemistry, Cambridge Centre for Proteomics, University of Cambridge, Cambridge, UK; 2 Celegene Institute for Translational Research Europe (CITRE), Sevilla, Spain; 3 Department of Computer Science, Computer Laboratory, University of Cambridge, Cambridge, UK

## Abstract

**Motivation:**

Protein–protein interactions (PPI) play a crucial role in our understanding of protein function and biological processes. The standardization and recording of experimental findings is increasingly stored in ontologies, with the Gene Ontology (GO) being one of the most successful projects. Several PPI evaluation algorithms have been based on the application of probabilistic frameworks or machine learning algorithms to GO properties. Here, we introduce a new training set design and machine learning based approach that combines dependent heterogeneous protein annotations from the entire ontology to evaluate putative co-complex protein interactions determined by empirical studies.

**Results:**

PPI annotations are built combinatorically using corresponding GO terms and InterPro annotation. We use a *S.cerevisiae* high-confidence complex dataset as a positive training set. A series of classifiers based on Maximum Entropy and support vector machines (SVMs), each with a composite counterpart algorithm, are trained on a series of training sets. These achieve a high performance area under the ROC curve of ≤0.97, outperforming go2ppi—a previously established prediction tool for protein-protein interactions (PPI) based on Gene Ontology (GO) annotations.

**Availability and implementation:**

https://github.com/ima23/maxent-ppi

**Supplementary information:**

Supplementary data are available at *Bioinformatics* online.

## 1 Introduction

Despite their structural diversity, proteins only achieve full potential by direct interaction in multi-protein complexes involved in fundamental biological processes such as gene expression, cell differentiation and cell–cell communication (Alberts, 1998; Bonetta, 2010; Vidal *et al.*, 2011).

Protein interactions have been studied by low-throughput assays and associated analytical methods, including x-ray crystallography (Scott *et al.*, 2009), nuclear magnetic resonance (NMR) and surface plasmon resonance (SPR), fluorescence resonance energy transfer (FRET) and isothermal titration calorimetry (ITC). Such methods are reviewed in (Collins and Choudhary, 2008; Shoemaker and Panchenko, 2007). Additionally, several mass spectrometry methods have more recently been used to interrogate protein interactions in multi protein complexes (Smits and Vermeulen, 2016). These structural proteomics approaches, including native mass spectrometry (Mehmood *et al.*, 2015), and crosslinking mass spectrometry (Liu *et al.*, 2015), nicely complement high-resolution cryo-electron microscopy (Huis In ’t Veld *et al.*, 2014).

The development of high-throughput approaches has generated large datasets, with the largest fraction being generated by yeast two-hybrid (Y2H) and affinity purification coupled with mass spectrometric identification (AP-MS) (Supplementary Note 1). These methods are not without limitations and false discoveries (Armean *et al.*, 2013; Deane *et al.*, 2002; Sprinzak *et al.*, 2003), despite experimental pipelines intended to reduce false interactions (Rees *et al.*, 2011, 2015).

### 1.1 Annotation ontologies

Computational methods to identify and filter false discovery from empirical output represent an alternative to assiduous and time-consuming experimental validation or use of simple subtraction of proteins from datasets based on their likelihood to be co-contaminants (Mellacheruvu *et al.*, 2013). An appropriate mapping between known properties of candidate proteins and their likelihood of interaction is key to the success of computational approaches.

In this context, many contemporary PPI prediction and evaluation algorithms use a range of associated information to describe likely binding partners, including co-expression and co-localization data, known involvement in biological processes, computational predictions of protein structure (Mosca *et al.*, 2013; Zhang *et al.*, 2012), and focused interaction data acquired using empirical approaches such as AP-MS (Armean *et al.*, 2013; Teo *et al.*, 2014).

Annotations that relate gene products to biological process, molecular function and sub-cellular localization have been curated for over a decade via the Gene Ontology (GO) (Ashburner *et al.*, 2000). Associated evidence codes describe whether annotations are derived from experimentation, computational analysis, author statements, during curation or by automated assignment (Rogers and Ben-Hur, 2009; Škunca *et al.*, 2012; Yon Rhee *et al.*, 2008). Each of the three ontology branches are hierarchically structured, with generic annotation terms, or nodes, forming roots for branches of more specific terms.

InterPro is a comprehensive database of protein domain annotations from more than a dozen databases (Mitchell *et al.*, 2015). The domain annotation is organized in a hierarchical structure, with domains that share higher-level structure and/or function at the top and those describing more specific functional subfamilies or structural/functional subclasses of domains at the bottom. Protein domains have been used in computational methods to identify PPIs either by single domain association (Sprinzak and Margalit, 2001), by frequency of domain co-occurrence or domain combinations (Han *et al.*, 2003). These methods are extensively reviewed by Ta *et al*. (Ta and Holm, 2009). Domain–domain interactions have been identified using 3D structures in PDB (Rose *et al.*, 2017), 3did (Mosca *et al.*, 2014) or predicted based on orthogonal information as PPIs with DOMINE v2.0 containing more than 20 513 known or predicted domain–domain interactions (Yellaboina *et al.*, 2011).

### 1.2 Prediction of protein interactions from annotation

Aside from the choice of classification algorithm, the availability of a realistic known or ‘training’ scenario that incorporates an appropriate annotation space within which to represent pairs of proteins is fundamental to such approaches. For a brief review of the training set design and GO based annotation space used in supervised machine learning applications to predict protein-protein interactions see Supplementary Note 2.

Most GO term similarity measures are restricted to descriptive probabilities of one shared GO term. There are multiple ways to select the most informative GO terms to compare: Jain and Bader used the first common ancestor (Jain and Bader 2010); Maetschke *et al.* (2012) compared an extensive list of approaches for selecting the parents concluding that the set of parents up to the first common ancestor is the most suitable (Maetschke *et al.* 2012), while Yang *et al.* (2012) used parents and descendants of the given GO terms to improve GO semantic similarity performance (Yang *et al.* 2012).

Many supervised machine learning approaches ignore some of the term relationships, therefore Maetschke *et al.* ( 2012) used similarity scores on all GO term parents up to the lowest common ancestor (ULCA), including both relationships (‘is_a’ and ‘part_of’) in a single random forest classifier. This approach was observed to perform better than (i) similarity scores applied to the most specific GO terms, (ii) similarity scores applied only to the lowest common ancestors (OLCA) or (iii) similarity scores applied to parent terms up to the lowest common ancestor (ULCA) excluding the lowest common ancestor itself (Maetschke *et al.*, 2012).

Boyanova *et al.* used the GO similarity of the Most Informative Common Ancestor (MICA) as implemented in the GOSim package (Fröhlich *et al.*, 2007) to build edge weights (Boyanova *et al.*, 2014). These edge weights in addition to node weights, based on presence/absence of proteins from reference networks, were grouped into specific functional modules by *heinz*, (heavy induced subgraph algorithm) (Dittrich *et al.*, 2008).

Methods to compute similarity scores between GO annotations have been grouped into node-based (GO terms), edge-based (GO term relationships) and hybrid methods, each with their own limitations (Pesquita *et al.*, 2009). Information Content (IC) sequence similarity is computed using the most informative node and the node’s use frequency, however the choice of node and the frequency can bias the results towards less studied species as their annotation frequency is lower than for more researched organisms. Edge-based methods, for example shortest path, are sensitive to terms with the same depth but different precision. Hybrid methods offer an alternative by defining the semantic similarity of one term as the sum of a chosen parent’s set (Pesquita *et al.*, 2009). A significant improvement to estimating semantic similarity using only child nodes or only parent nodes is to use both sets to infer similarity. The combination of both sets raises the question of how to best use the ontology structure to maximize inference (Bettembourg *et al.*, 2014; Mazandu and Mulder, 2013).

Co-evolution of proteins, the presence/absence of protein pairs across taxa, has been used in several similarity scores and made available through STRING (Szklarczyk *et al.*, 2015), Prolinks (Bowers *et al.*, 2004) or ECID (Andres Leon *et al.*, 2009). These methods range from using binary representation to a mix of similarity metrics and taxonomy. A recent development is an automated approach, sub-setting the taxa to the most informative set of species for the specific organism PPI prediction problem (de Juan *et al.*, 2013; Ochoa and Pazos, 2014; Simonsen *et al.*, 2012; Škunca and Dessimoz, 2015).

The combination of protein phylogenetic profiling, phyloprof (Simonsen *et al.*, 2012) and go2ppi (Maetschke *et al.*, 2012) resulted in an improvement from 0.61 to 0.7 AUC (area under the ROC curve) when trained and tested on yeast PPI published data (Yao *et al.*, 2015). More recently the information content of a GO term for a specific protein has been adjusted based on all the other GO terms, including ancestors terms, assigned to the protein (Bandyopadhyay and Mallick, 2017). All GO ancestor terms have been recently used as part of a new Weighted Inherited Semantic (WIS) measure (Tian *et al.*, 2016).

### 1.3 Maximum Entropy

Maximum Entropy modelling is considered to be among the simplest predictive models, as its only constraint is to train a model that maximizes expected disorder in the system as quantified by the entropy. Information theory and Maximum Entropy were successfully used by Alterovitz *et al.* (2010) to evaluate and suggest improvements to the GO ontology structure based on terms at the same depth level having varying information content, inter-level variability (one parent-child relationship might encode a higher information content increase than another) and topological variability (Alterovitz *et al.*, 2010). The knowledge gained in respect of the GO ontology was used to create an improved GO: single-level changes were introduced, 1001 relationships and 11% of GO terms modified. The modifications lead to a significant change in functional interpretation for 97.5% of genes and on average 14.6% of GO categories.

Here, we introduce a novel approach that uses Maximum Entropy to capture and take advantage of the entire ontology structure—all relationships and ancestor terms—that offers an efficient method for protein co-complex evaluation with insight into the individual weights for each annotation. We apply a Maximum Entropy model, GIS-MaxEnt [Generalized Iterative Scaling Maximum Entropy (Darroch and Ratcliff, 1972; Jain *et al.*, 2005)] to the interaction prediction scenario described above and assess its predictive power.

## 2 Materials and methods

### 2.1 Problem formulation and dataset

**Fig. 1.**
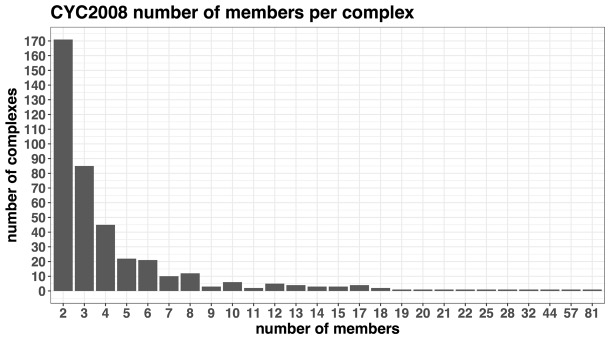
Barplot distribution of the number of members per complex in the CYC2008 dataset of 408 complexes. The four largest complexes are: the cytoplasmic ribosomal large subunit with 81 members, the cytoplasmic ribosomal small subunit with 57 members, the mitochondrial ribosomal large subunit with 44 members and mitochondrial ribosomal small subunit with 32 members. Most complexes 171/408 (42%) have 2 members

The manually curated *S.cerevisiae* CYC2008v2.0 (Pu *et al.*, 2009) dataset was selected as a starting point when building a set of high confidence protein interactions (true positives). The CYC2008v2.0 dataset comprises 408 manually curated protein complexes obtained from consolidation of two genome-wide affinity purification mass spectrometry (AP-MS) studies. The number of complex members ranges from 2 to 81 (cytoplasmic ribosomal large subunit) with a median of 3 complex members (Fig. 1). This dataset has been used as a validation set for the study of conservation of multiprotein complexes among metazoans (Wan *et al.*, 2015) and in the identification of essential proteins based on PPI networks and complexes (Qin *et al.*, 2016).

Matrix expansion—a method that assumes binary interaction between any bait–prey or prey–prey proteins identified in the same experiment—was used to expand the 408 high-confidence overlapping complexes to 11 923 *S.cerevisiae* binary interactions among 1627 genes, of which 211 belonged to more than one complex. An alternative to matrix expansion, that we chose not to employ, is the more conservative spoke expansion whereby bait proteins are assumed to interact only with prey proteins (Hakes *et al.*, 2007).

Considering the rate at which proteins are annotated [estimated 300–500 proteins in 6 months (Radivojac *et al.*, 2013)] it is sensible to expect that annotations were assigned based on the publication of this dataset. Using annotations created due to the publication of a dataset when evaluating the same dataset would result in circularity and bias in the model. To avoid this bias, the 11 923 *S.cerevisiae* interactions were transferred by homology to *D.melanogaster* interactions. The mapping was performed by identification of interologs of yeast (Walhout, 2000) in Drosophila. The gene homologs were extracted using FlyMine v. 33 (Lyne *et al.*, 2007) which includes TreeFam v7.0 (Ruan *et al.*, 2008). The 11 923 *S.cerevisiae* interactions were transferred to 9593 binary interactions among 1077 genes in *D.melanogaster*. These 9593 binary interactions are considered to have high confidence, and hence form the positive set.

In order to create a negative training set counterpart, 9593 pairs of genes were randomly sampled from the set of 1077 genes, ignoring pairs of genes already present in the positive set or published as interacting based on FlyMine v33, which imports BioGRID (Stark *et al.*, 2011), IntAct (Kerrien *et al.*, 2012) and FlyBase (McQuilton *et al.*, 2012). This approach ensured the same level of protein annotation in both training classes. Additional filters including different subcellular locations (Jansen *et al.*, 2003) were later assessed as introducing significant bias into the training problem (Ben-Hur and Noble, 2006). Depending on organism and model, GRIP (Browne *et al.*, 2009) and Negatome (Smialowski *et al.*, 2010) offer alternative approaches for training set construction.

The gene pairs present in the positive and negative set will be referred to as protein interactions in the remainder of this work.

### 2.2 Annotation

GO and InterPro annotations including all parent terms were extracted for each gene using FlyMine v33. GO terms with evidence codes NAS (Non-traceable Author Statement), ND (No biological Data available), IEA (Inferred from Electronic Annotation) and NR (Not Recorded), or, those labelled with the ‘Not’ qualifier, were excluded. Given individual GO branch depths, most unique annotation pairs originated from the biological process branch (Supplementary Table S1). 841 of the 1077 genes had at least one GO or InterPro annotation (Supplementary Table S2 for gene annotation coverage).

The distribution of the shortest paths from each GO term to its corresponding root is slightly skewed towards shorter distances (Supplementary Fig. S1). For biological process (BP) and molecular function (MF) most of the terms are centered half way down the ontology tree.

### 2.3 Annotation representation

Protein annotation was transferred at the interaction level by pairing all annotation terms ($P_{1}=\{GO_{1x}|x=1..n\}$), including all parents, from one protein with all annotation terms ($P_{2}=\{GO_{2y}|y=1..m\}$) from the other protein such that $P_{1}P_{2}=\{GO_{1x}GO_{1y}|x=1..n,y=1..m\}$. The three GO branches were treated separately.

**Fig. 2.**
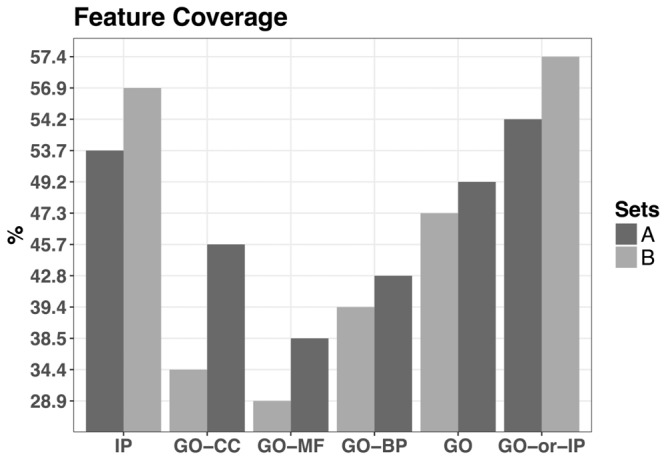
Annotation coverage of the protein interactions of the two initial training sets each containing 9593 interactions

The above approach resulted in the annotation coverage of the protein interactions being 54.25% (5204/9593) for the positive set (A) and 57.42% (5508/9593) for the negative set (B) (Fig. 2 and Supplementary Table S3).

By using all GO parent terms in our annotation preparation, we ensure that the root terms will most often form pairs leading to high frequency in observance, and a low information content evaluation by the Maximum Entropy model, as well as ensuring that any relation between the child and parent terms is maintained.

In the positive set (A) 3164 gene pairs had at least one annotation in each of the annotation categories GO-CC, GO-MF, GO-BP and InterPro (IP). Out of these 3164, 903 (28%) were interactions involving histone proteins, 880 (27%) were ribosomal protein interactions related to the large ribosomal subunit, and 522 (16%) related to the small ribosomal subunit (Supplementary Fig. S2). To avoid sampling a large number of ribosomal or histone interactions, which would have made the positive subset very specific, these interactions were excluded before sampling. After exclusion of histone and cytoplasmic ribosomal related proteins, the positive set comprised 859 interactions. For ease in performance testing, 500 out of the 859 examples were randomly sampled.

The structure of the ontologies is reflected in the number of unique annotations extracted from each of them; the highest number of annotations is present in the GO biological process branch (22 259 over 15 levels), while InterPro has fewer terms (6622 with the maximum depth of 8 levels). The number of annotations obtained from each annotation source in the final training set is displayed in Supplementary Table S4. The negative set covers a broader range of annotations than the positive set, due to the increased randomization of the data.

### 2.4 Classifiers

We used four machine learning methods to predict PPIs. Generalized iterative scaling maximum entropy (GIS-MaxEnt) (Darroch and Ratcliff, 1972) and support vector machines (SVMs) (Shawe-Taylor and Cristianini, 2004) are standalone methods. The other two methods—GIS-MaxEnt Ensemble and Multiple Kernel Learning (MKL)—are combinations of classifiers. A brief introduction to the underlying algorithms is given in the Supplementary Data, along with details of the specific software used. This section provides information that is specific to our own experiments.

For the two standalone classifiers we trained on each individual annotation source GO-BP, GO-CC, GO-MF and IP. We also used two combined sources: GO, which combines the GO-BP, GO-CC and GO-MF sources, and GO-IP which includes all the sources combined. The GIS-MaxEnt Ensemble and MKL methods were allowed to combine GO-BP, GO-CC, GO-MF and IP as part of the training process.

#### *2.4.1* Generalized iterative scaling—maximum entropy

Internally the GIS-MaxEnt method specifies the feature functions $f_{i}\left(x,y\right)\;:\;X\;\times \;Y\to \left\{0,\;1\right\}$ that act on training examples (x,y). In our experiments using the kernel methods we used the feature functions

fix,y=1, if GO term pair i is in x,y0, otherwise.                                

The implementation of GIS-MaxEnt used (Supplementary Note 3) was modified to use the mean number of annotations per interaction as internal correction constant in the training step, as opposed to the maximum number of annotations. Subsequent to this we employed the default settings with a maximum of 100 iterations.

#### *2.4.2* Support vector machine (SVM)

We employed a kernel K derived from the polynomial kernel (Shawe-Taylor and Cristianini, 2004)

$$K\left(x,x^{'}\right)=\;{\left(〈x,x'〉+c\right)}^{d}$$

where $〈x,x'〉$ denotes the inner product of x and $x^{'}$. Specifically we set c=0 and d=1 and refer to the kernel $K\left(x,x^{'}\right)=〈x,x'〉$ as the *linear kernel*.

A common preprocessing step when applying machine learning methods is to normalize the datasets such that features have a mean of zero and a standard deviation of one. However, the datasets described above are both large and sparse, and normalizing the features would make them dense. Instead of doing this we implemented a kernel normalizer to normalize by the Tanimoto coefficient (Tanimoto, 1958). This operates directly on the kernel matrix, which is significantly smaller than the feature matrices. It computes the Jaccard similarity as

$$K'\left(x,x^{'}\right)=\;\frac{K\left(x,x^{'}\right)}{K\left(x,x\right)+K\left(x',x^{'}\right)-K\left(x,x^{'}\right)}$$

where K' is the normalized kernel, K is the original kernel—the linear kernel in our experiments—and x and x' are feature vectors.

#### *2.4.3* GIS-MaxEnt ensemble

Individual GIS-MaxEnt models were trained on the four data subsets and their resulting predictions on the training data used as input to a linear SVM decision layer (Supplementary Fig. S3).

#### *2.4.4* Multiple kernel learning

A kernel ${K'}_{i}$ was constructed for each of the data subsets, based on the Jaccard similarity and linear kernel as was the case for the SVM. The multiple kernel was then

$$K''\left(x,x'\right)=\;\sum_{i=1}^{4}\beta_{i}{K'}_{i}\left(x,x'\right)$$

We used the $\ell_{2}$-norm on the values $\beta_{i}$ (Supplementary Note 4).

### 2.5 Model selection and estimated generalization

Estimated generalization performance was assessed by repeated stratified (Stratification in this context refers to the preservation of the original class proportions in each partition.) partitioning of all labelled data examples into training (90%) and test (10%) partitions. Models were created on the training partition and their predictions assessed on the test partition. We used 50 divisions of the data to assess each method (more details in Supplementary Note 5 and Fig. S7).

### 2.6 go2ppi system

Maetschke *et al.* compared 10 different approaches of generating a set *S* of GO terms based on two sets of GO terms *S1* and *S2*, each corresponding to different proteins. The ULCA approach outperformed the others and was selected to be implemented in the go2ppi system. The GO term set *S* for each protein interaction was transformed to a binary feature vector *v* where each unique GO term corresponded to a unique index *i* and the corresponding position in the vector was set to 1 if the GO term was present or 0 otherwise. Using this configuration, a sparse high‐dimensional matrix was built and used as input to a machine learning algorithm. The go2ppi software offers two algorithm implementations: Naïve Bayes (NB) and Random Forest (RF) (Maetschke *et al.*, 2012).

The go2ppi system is an appropriate system for comparing against the proposed ontology-based models. First, there is an extensive list of approaches for extracting PPI relevant GO terms explored by the go2ppi authors with the one performing best being implemented in go2ppi. Secondly, the go2ppi pipeline is freely available.

The labelled examples were used to create two input files as required by go2ppi: a binary protein—protein interaction file and a protein annotation file. The annotation file containing only the most specific GO terms. go2ppi (version 1.06) was set to be evaluated with 10-fold cross validation, 90%/10% split and 50 runs to replicate the training and testing of our own methods. The same GO obo (open biomedical ontologies format) version was used as in FlyMine v33, and both Random Forest (RF) and Naïve Bayes (NB) implementations were tested. go2ppi reports the AUC in the training phase and testing phase.

## 3 Results

### 3.1 Performance comparison: GIS-MaxEnt versus SVM

The GIS-MaxEnt and SVM models’ performance was assessed on a *D.melanogaster* training set composed of 500 positive examples and 500 negative examples described by 224 629 annotations based on InterPro and GO annotation terms (see Supplementary Table S4 and Section 2).

#### *3.1.1* GIS-MaxEnt applied on different annotation sets

**Fig. 3.**
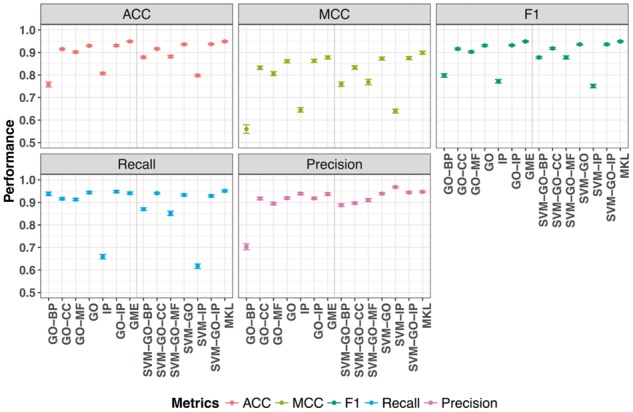
Performance of the different systems trained on the different datasets evaluated using accuracy (ACC), Matthews Correlation Coefficient (MCC), F1, recall and precision as defined in the formulas (Supplementary Tables S5, S6). GIS-MaxEnt trained on the six different training sets: GO-BP, GO-CC, GO-MF, GO, GO-IP, SVM trained on the same six training sets: SVM-GO-BP, SVM-GO-CC, SVM-GO-MF; SVM-GO, SVM-IP, SVM-GO-IP; GIS-MaxEnt Ensemble (GME) and Multiple Kernel Learning (MKL) which were trained on all the data

The GIS-MaxEnt based model trained on the four individual data sources (three GO branches and one InterPro) performed best when trained on the GO *cellular component* having a Matthews correlation coefficient (MCC) of 0.83 with the lowest performance being present for the one trained on *biological process* with a MCC of 0.56 (Fig. 3 and Supplementary Table S6). A reduced number of annotations does not correlate directly to a lower MCC, for example there were 8632 InterPro based terms and 8875 GO *molecular function* terms (Supplementary Table S4) resulting in 0.64 MCC for InterPro and 0.80 for the GO branch. The observed difference is likely due to the underlying ontology structure with the *biological process* GO branch having the most terms and the maximum number of levels. The InterPro structure is shorter in depth (maximum 8 levels deep) and very wide (1926 distinct terms on the first level) reflecting the diversity in protein families. The difference between the individual datasets is also highlighted when plotting the ROC curves (Supplementary Fig. S4).

Varying accuracies were obtained on the individual datasets, with the combination of all four leading to the highest accuracy of 0.93 and the highest AUC of 0.979 (Fig. 3).

The GIS-MaxEnt model trained on all three GO branches also has a very good performance and is not significantly different to the performance on the GO-IP dataset [Wilcoxon unpaired two sample test, *P*-value < 0. 05 (Supplementary Table S7)]. We observe the difference between the performance of GO-CC and GO-MF to be less significant than the one between GO-CC and GO-BP or GO-CC and GO-IP. Regardless of its low number of annotations (Supplementary Tables S1, S2) the GO-CC branch is the second dataset in respect of performance contribution to the GIS-MaxEnt GO-IP dataset.

#### *3.1.2* SVM applied on different annotation sets

The SVM has a high performance (AUC above 0.8) on all of the training sets, with the GO-IP dataset having the highest AUC 0.984 (Supplementary Fig. S5). Based on MCC, SVM-IP is the least successful combination, with SVM-BP and SVM-GO-MF being relatively comparable and SVM-GO-CC having a higher MCC of 0.83 (Fig. 3 and Supplementary Table S6).

The slight increase of SVM-GO-MF MCC over SVM-GO-BP is overturned when the AUC is taken into consideration, however the performance difference is not significant (Supplementary Table S8). Any of the trained SVM models display a significant performance difference against SVM-GO-IP except SVM-GO which has *P*-value = 0.68 (Wilcoxon test on MCC values).

#### *3.1.3* GIS-MaxEnt compared to SVM

GIS-MaxEnt and SVM perform well on the different training sets. There are some notable differences. If the MCC performance ranking of the four primary datasets (GO-MF, GO-BP, GO-CC, IP) for each method is compared then the only datasets that do not change position are GO-CC and GO-MF, being ranked first and second. Only GO-CC maintains its rank when also taking the AUC into consideration.

GIS-MaxEnt maintains the performance rank between MCC or AUC, while SVM has an inversion of the rank for GO-BP and GO-MF, which is not surprising given the relatively small difference in MCC performance.

Comparing the two trained models GIS-MaxEnt and SVM to each other on the same sets, they have a significantly different performance for GO-BP set (*P-*value 9.29E-11), GO *biological process* ontology being the one with the highest number of terms. If the *P*-value is taken as a measure of similarity, then the models trained on IP are most similar, followed by GO-CC, suggesting that the InterPro and *cellular component* annotations are able to clearly separate the positive from the negative examples and therefore represent good quality annotation.

The significantly different performances between the models on the other datasets suggests that each model has learned different separation rules from the same training set, despite similar performance (Supplementary Table S9).

### 3.2 Ensemble classifiers

#### *3.2.1* GIS-MaxEnt compared to GIS-MaxEnt ensemble

The ensemble version of GIS-MaxEnt has a slight improvement over GIS-MaxEnt trained on GO-IP in respect of AUC, from 0.979 to 0.981 (Fig. 3, Supplementary Fig. S6) although the difference when compared on MCC is not significant (Wilcoxon test, *P*-value < 0.05, see Supplementary Table S10).

GIS-MaxEnt trained on InterPro compared to the GO based sets continues to have the lowest *P-*values (*P-*value 1.34E-17 see Supplementary Table S8, *P-*value 1.49E-17 see Supplementary Table S11). This reflects the significantly different annotation structure of the InterPro annotation vocabulary compared to the GO.

#### *3.2.2* GIS-MaxEnt ensemble compared to MKL

Analysis presented in Table 1 show that MKL is in agreement with GIS-MaxEnt when trained on individual sources, in evaluating GO-BP as having the lowest contribution to the overall evaluation and GO-MF the second highest. However they disagree regarding the top-ranked contributor: InterPro or *cellular compartment* (Table 1, Supplementary Table S6).

**Table 1.** The individual weights on each dataset used by the MKL algorithm

GO-CC	GO-BP	GO-MF	IP
0.428±5E-05	0.422±3E-05	0.538±7E-05	0.590±11E-05

In both the GIS-MaxEnt and kernel-based systems, the algorithms trained on all the data sources outperform models trained on individual sources alone. The ensemble models outperform the models trained on all data at once (Supplementary Table S7). The MKL and GIS-MaxEnt Ensemble had almost identical performance with MKL having a slightly improved MCC (Supplementary Table S6) although the difference was not significant (Wilcoxon test *P*-value < 0.05).

### 3.3 Performance in the context of published systems

Compared to go2ppi, GIS-MaxEnt had higher AUC performance both when using only the most specific terms and when including the GO parent terms, outperforming both go2ppi configurations using either Naïve Bayes or Random Forest (Table 2).

**Table 2.** AUC for go2ppi and GIS-MaxEnt in different configurations

Model	GO-CC	GO-BP	GO-MF	GO
go2ppi—NB	0.765/0.730	0.731/0.700	0.729/0.697	0.761/0.723
go2ppi—RF	0.991/0.719	0.985/0.697	0.957/0.695	0.997/0.708
GIS-MaxEnt —term-only	0.963	0.959	0.950	0.972
GIS-MaxEnt —all-parents	0.965	0.787	0.956	0.978

*Note*: The go2ppi algorithm reports two results, displayed as Train/Test. ‘Train’ is the self-test AUC in the training phase (for example 0.731 for go2ppi-NB and GO-BP). ‘Test’ is the 10-fold cross-validation AUC in the testing phase over 50 runs (for example 0.70 for go2ppi-NB and GO-BP).

The performance of GIS-MaxEnt is highest when all the GO branches are used, with a slight improvement when the parent terms are used as opposed to term-only (Table 2). The ranked order based on performance is maintained with GO-CC having the highest performance followed by GO-MF and GO-BP. The ontology branch GO-BP has more than twice as many terms as GO-MF distributed over the same number of levels (Supplementary Table S1). This has an impact on the performance of GO-MF and GO-BP in the two modes: term-only and all-parents. The model trained on GO-BP term-only is overfitting and GO-BP all-parents has a lower performance having to deal with a lot more terms from the dense structure. This is also reflected in the very large number of annotations obtained when using all the parents (Supplementary Table S4). This property of the GO-BP branch leads to parent term-based annotation having a higher overlap between leaf terms belonging to a positive example of PPI and a negative example of PPI, making the separation between positive and negative examples harder, although at the same time one could expect that this property could give the evaluation a better resolution. Based on the difference of 0.78 versus 0.95 for GIS-MaxEnt and GO-BP it is expected that the GIS-MaxEnt (term-only) model is overfitting, having a very good performance on the training set and limited performance on new examples, due to unseen combinations of GO-BP terms. GO-CC maintained the top ranked dataset for the Naïve Bayes and Random Forest models, followed by GO-BP and GO-MF.

The self-test AUC is always higher than the testing phase as it is computed on the same dataset as the training (Table 2). The RF displays a bigger difference between the self-test AUC and 10-fold cross-validation AUC suggesting that Random Forest is more prone to overfitting than the Naïve Bayes model.

### 3.4 GO term frequencies

**Fig. 4.**
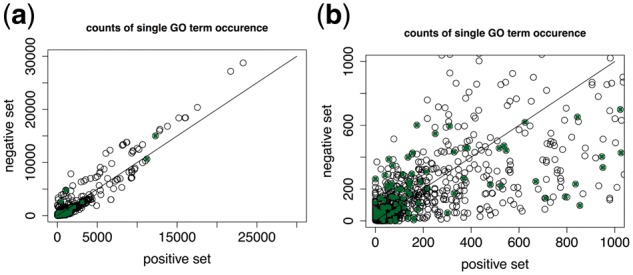
Plot of occurrences of GO terms defining protein complexes (full dots) in the positive and negative set compared to the rest of the GO terms (empty circles). The difference in counts of all GO terms between the positive and negative set is significant at *P* < 0.05 (*P*-value = 0.028), while the frequencies for the 180 protein complex GO terms do not differ significantly (*P*-value = 0.28). The right plot (b) is a closer view of the points in the 0 to 1000 range (a)

Both GIS-MaxEnt and MKL performed well on the training set. To check that the problem setting and training set did not represent a trivial question for the algorithms, we looked for the presence of GO terms representing protein complexes in our training set. 1679 GO terms were extracted from the GO (v1.1.2412) containing the word ‘complex’ in the name. Out of these 180 were present in the training set, and the counts in the positive set were not significantly different from those in the negative set (Wilcoxon test *P*-value = 0.28). The frequencies of all single GO terms in the positive and negative set are however significantly different at *P*-value < 0.05 (Fig. 4).

As an independent validation we evaluated the 359 positive PPIs excluded from the training set by random selection (Section 2.1) using GIS-MaxEnt trained on GO-IP. 95.8% (344 out of 359) were correctly evaluated with a score above 0.5.

In addition to an efficient classification GIS-MaxEnt offers the insight into the individual weights assigned to term pairs present in the positive and negative training set (Supplementary Fig. S8).

To further assess the performance of the GIS-MaxEnt based system, we assessed it using a recently published dataset containing 1379 binary interactions in *S.cerevisiae* (Celaj *et al.*, 2017). Unlike the Drosophila dataset where interactions were determined using affinity purification coupled with mass spectrometry, this yeast interaction dataset was created using a murine dihydrofolate reductase protein complementation assay (mDHFR PCA). The resulting binary yeast protein interactions were detected in at least one out of 14 different biochemical conditions with many being condition dependent (55%). Using the 1379 interactions as a positive training set, we created a corresponding negative set and annotated it with GO annotations as described in the methods section (section 2, Supplementary Note 6). Based on 10-fold cross-validation the highest accuracy was obtained when all three ontology branches in GO were used, giving an accuracy value of 0.84 with a recall of 0.87 and precision of 0.82 (Supplementary Table S12).

Comparing the results from the two datasets shows the GIS-MaxEnt performance is better on the more highly curated, Drosophila dataset. Nevertheless, the good performance on the *S.cerevisiae* set showcases the direct applicability of our system to protein interaction sets obtained with different experimental methods beyond AP-MS in spite of differences in curation.

## 4 Discussion

In this work we set out to design and test a novel PPI evaluation system. We created a novel training set for *D.melanogaster* based on published curated *S.cerevisiae* protein complexes from the CYC2008 dataset, revisiting data representation and training set design. We have evaluated the combination of an information theoretic algorithm with protein annotation databases to assess experimentally derived protein interactions. The training set was complemented by a novel way of using controlled vocabulary annotation stored in ontology structures. We tested the performance of several algorithms on the novel training set and annotation representation and obtained good estimated generalization performance and good performance when applied to a larger test set.

The GIS-MaxEnt and SVM models trained on the merged dataset containing all individual sources outperform GIS-MaxEnt trained on any of the individual sources. The GIS-MaxEnt Ensemble and MKL outperform their counterparts trained on GO-IP, with MKL having a slightly improved MCC compared to the GIS-MaxEnt Ensemble, however the performance comparison did not pass the significant threshold. To conclude, both algorithm types performed well, but by learning different rules had occasionally significantly different performance.

The GIS-MaxEnt based system was also compared against the publicly available go2ppi system, which made use of its own approach to building the parent GO terms set (Up to Lowest Common Ancestor ULCA). This approach of selecting the GO parents outperformed an extensive variation of methods of obtaining GO parents. The GIS-MaxEnt system outperformed both the go2ppi implemented algorithms—Naïve Bayes and Random Forest—when trained either on GO specific terms only or GO including all parent terms.

The model using all GO parent terms offers an improved discrimination of PPIs compared to using only the most specific GO terms, this being due to the higher number of GO based annotations that the model was trained upon. A similar trend can be observed, based on the GIS-MaxEnt AUC performance, for the GO term-only model versus the GO all-parent-terms model. However, despite the lower AUC value when using GO all-parents, this model highlights a central property of the underlying ontology: that the GO-BP ontology has a very dense branching system, with the result that positive and negative PPIs share many of the GO parent terms. A high number of shared GO parents between the positive and negative PPIs will lead a model to assign less extreme weights to the shared GO term based annotations. However, the GIS-MaxEnt based system outperformed one of the latest developments in GO based PPI evaluation (Maetschke *et al.*, 2012) even when used only on GO specific terms.

The proposed approach is limited to the annotation terms observed in the training set. The use of only the most specific annotated GO terms is likely to lead to overfitting and poor performance on unseen annotation. The use of species-specific training sets ensures that only the species relevant ontology space will be trained and therefore reduce the likelihood of missing important unseen annotation. The proposed system however performs well in the context of increased usage of ontologies and standardized controlled vocabularies.

Here we showcased the application of GIS-MaxEnt on categorical annotations. Continuous numerical annotation, such as interaction weights, could be readily usable by representing them into a categorical system.

To conclude, we introduce a novel approach to the computational quality assessment of protein interaction screens and a novel training set for evaluating protein complex data in *D.melanogaster*. This system has been trained and applied on a large dataset, which is part of the FlyTrap project (Lowe *et al.*, 2014) and accessible through FlAnnotator (Ryder *et al.*, 2009). Transparent evaluation of PPIs and the individual weights for the annotation term pairs will support further ontology refinement and PPI analysis as part of powerful aggregate systems such as STRING (Szklarczyk *et al.*, 2015).

## Acknowledgements

We would like to thank Iain Bancarz for constructive discussions before the start of this project while at EMBL-EBI.

## Funding

I.M.A. was funded by the Biotechnology and Biological Sciences Research Council [BBSRC grant BB/F017464/1].

*Conflict of Interest:* Since direct involvement in this work, MWBT has become an employee of Celgene Research SL, part of Celgene Corporation and declares no conflict of interest.

## Supplementary Material

Supplementary Data
