# Supplementary material for: Co-complex protein membership evaluation using Maximum Entropy on GO ontology and InterPro annotation
Source: Bioinformatics. 2018 Jan 30;34(11):1884–92. doi: 10.1093/bioinformatics/btx803 (PMC5972588; doi:10.1093/bioinformatics/btx803)
Supplement: Supplementary Data [file btx803_bioinformatics_armean_supplementary_re_submit.doc]

Supplementary data

1. Figures:


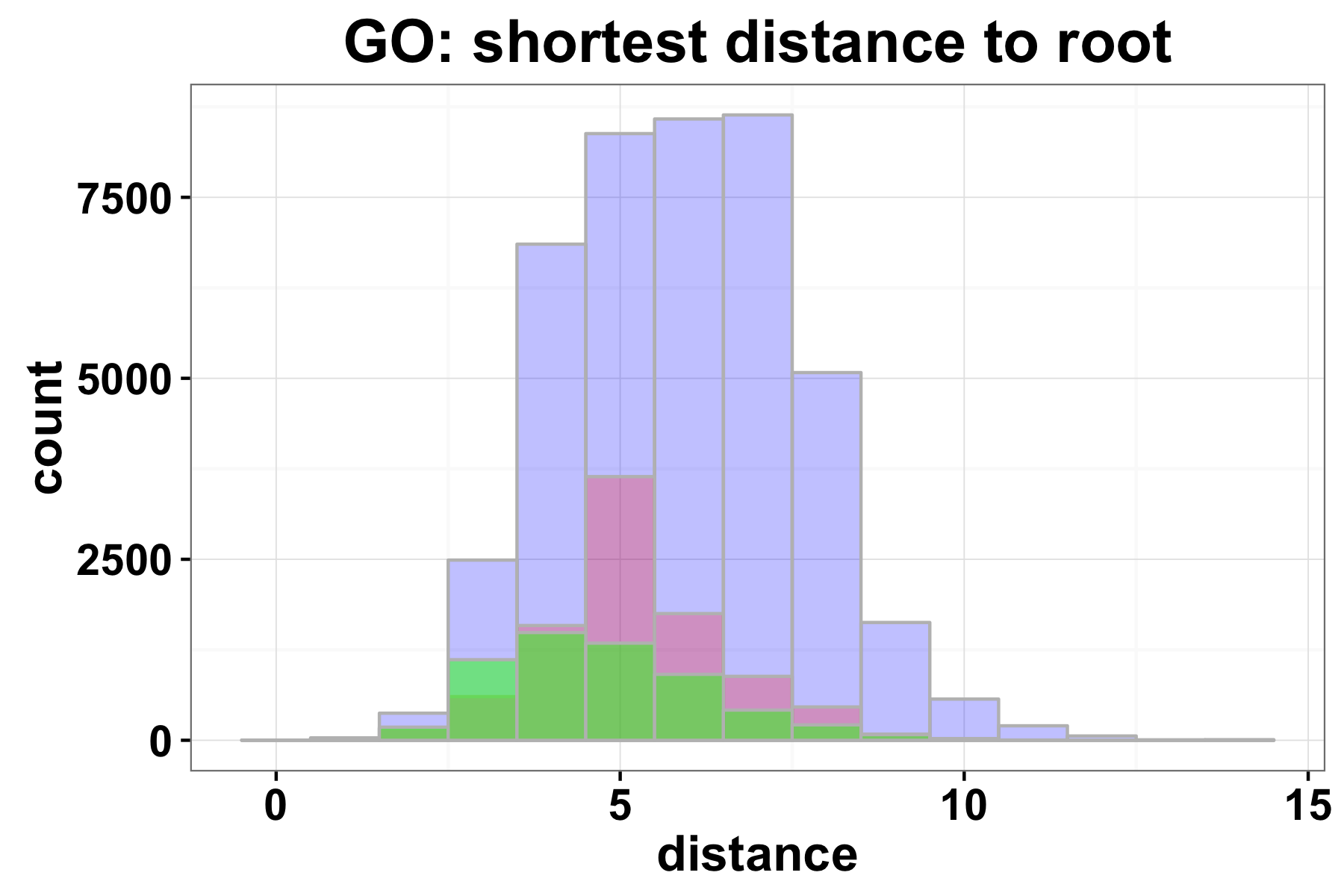


**Figure S1**. Histogram of the shortest distances for each GO term to the corresponding GO term root (biological process – light blue, molecular function – light red, cellular component – light green)


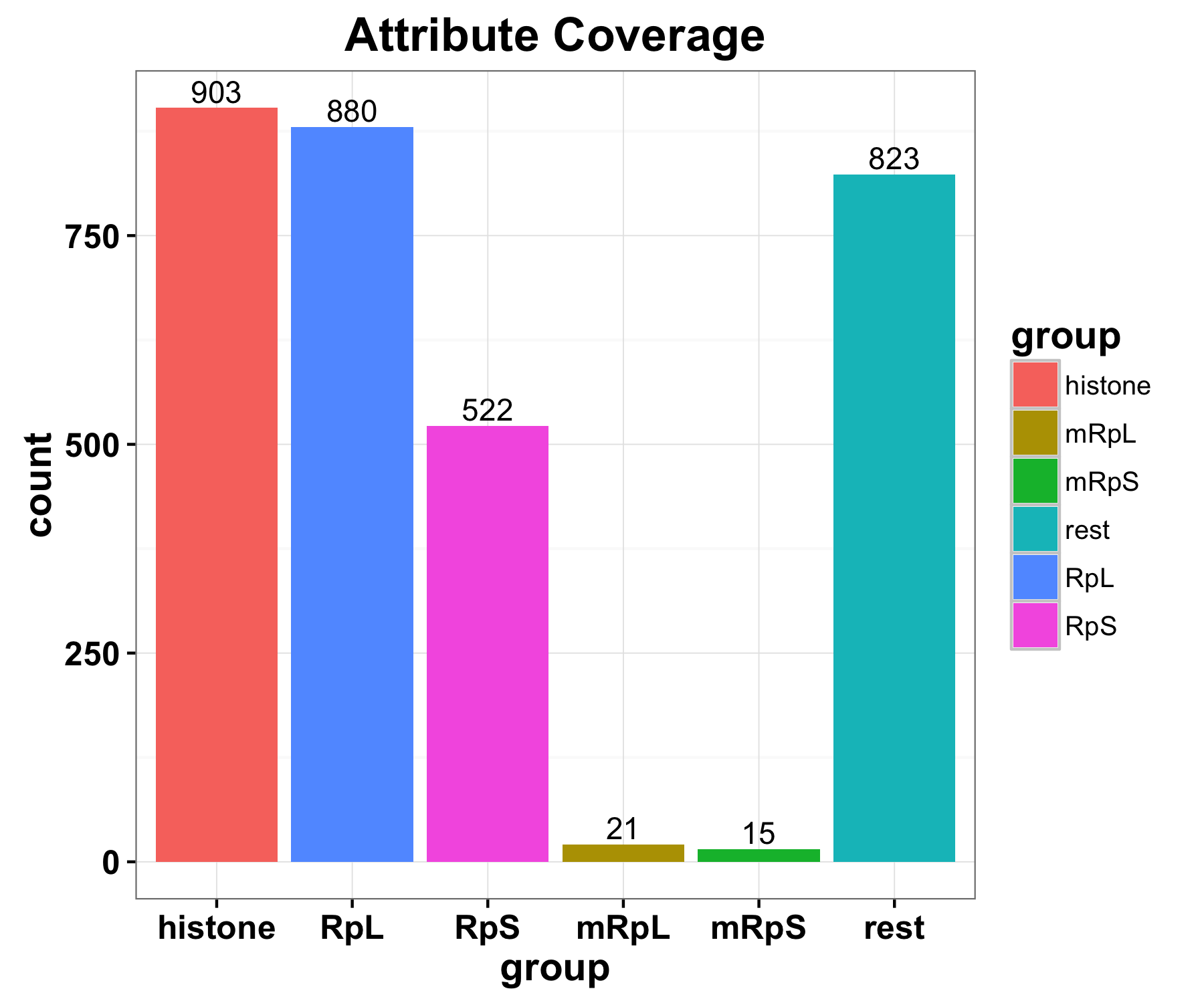


**Figure S2.** Representation of complexes in the 3,164 positive set. RpL: cytoplasmic ribosomal proteins in the large subunit; RpS: cytoplasmic ribosomal proteins in the small subunit; mRpL: mitochondrial ribosomal proteins in the large subunit; mRpS: mitochondrial ribosomal proteins in the small subunit.


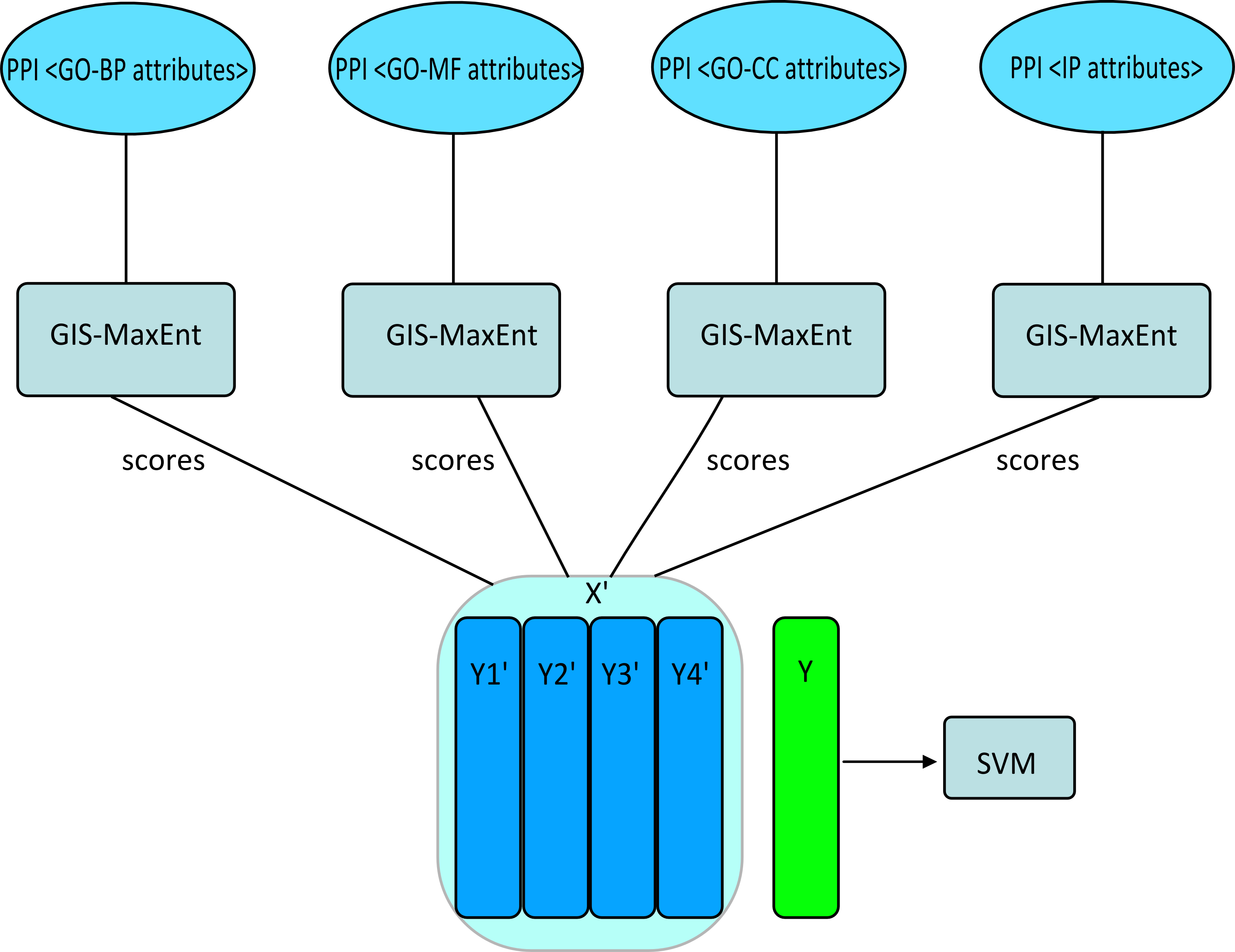


**Figure S3**. Graphical representation of the ensemble model obtained by applying SVM as a decision layer to the GIS-MaxEnt scores obtained from each annotation source dataset.
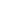
 denotes the input matrix for the SVM and
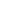
 the output, with
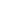
,
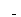
,
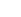
,
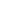
 being the output from GIS-MaxEnt model trained and evaluated on each individual annotation source.


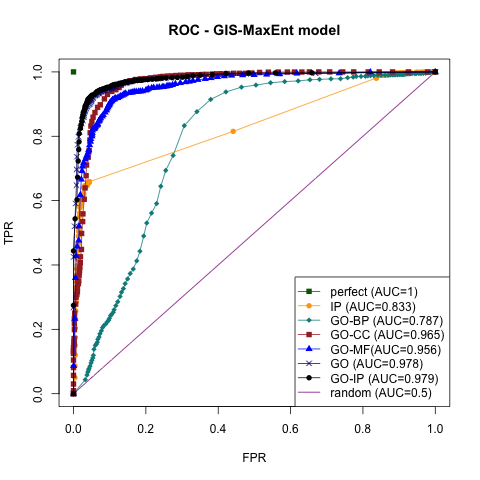


**Figure S4.** ROC curves for each trained model using GIS-MaxEnt in comparison to a random model (random) and a perfect classifier (perfect). The figure shows that the use of all annotations has the highest AUC of 0.979. FPR: false positive rate, TPR: true positive rate.


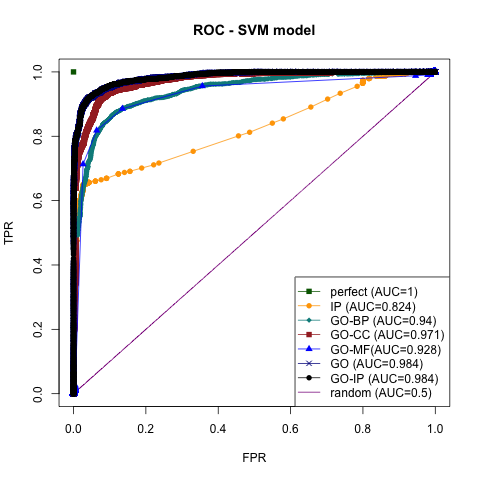


**Figure S5.** ROC curves (5000 points) for each trained model using an SVM in comparison to a random model (random) and a perfect classifier (perfect). The highest AUC of 0.984 is achieved by an SVM trained on the GO-IP dataset. FPR: false positive rate, TPR: true positive rate.


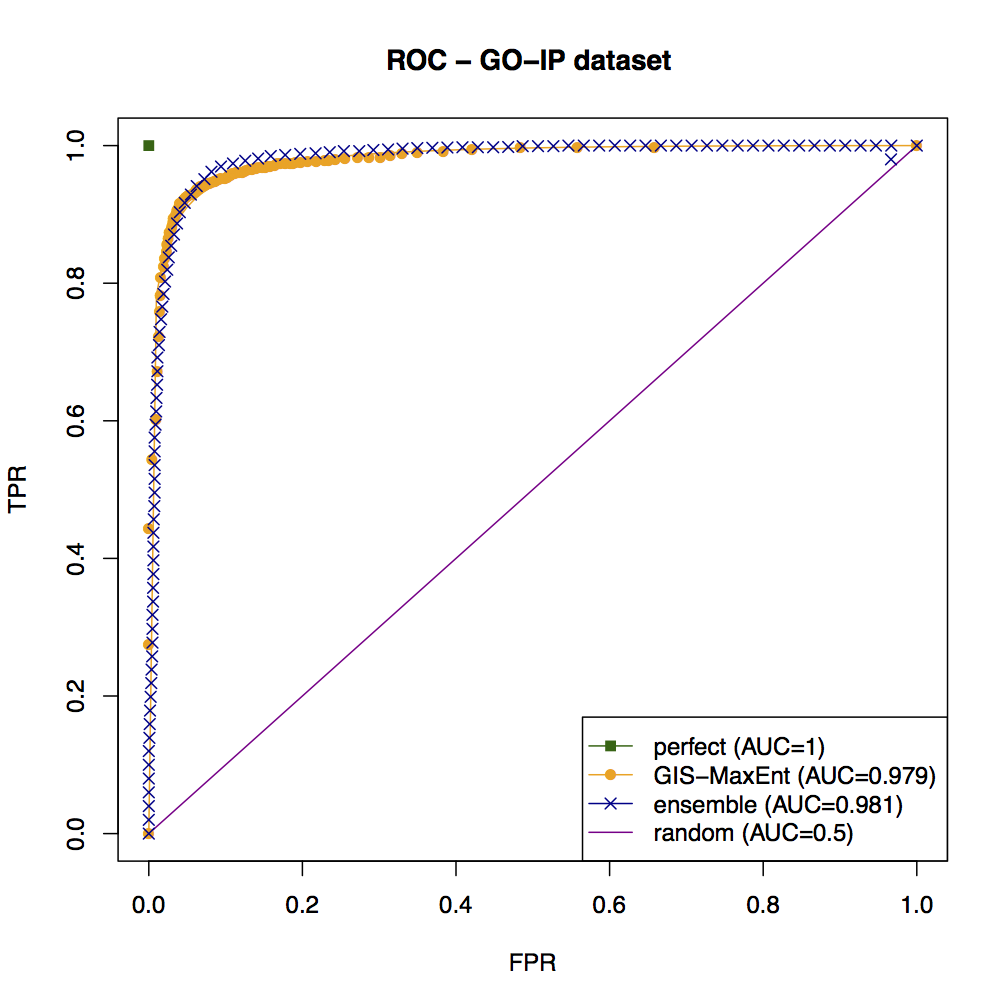


**Figure S6.** ROC curve comparison between the GIS-MaxEnt trained on GO-IP (orange) and the ensemble version of GIS-MaxEnt - an SVM trained on the results of GIS-MaxEnt on each data set (dark blue).


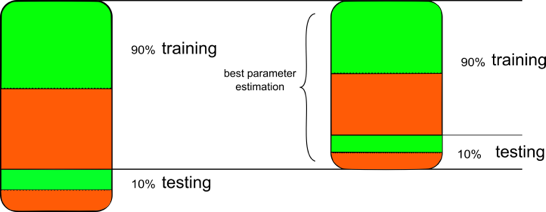


**Figure S7.** 10-fold cross validation using internal loop for optimal parameter selection using 50 internal partitions.


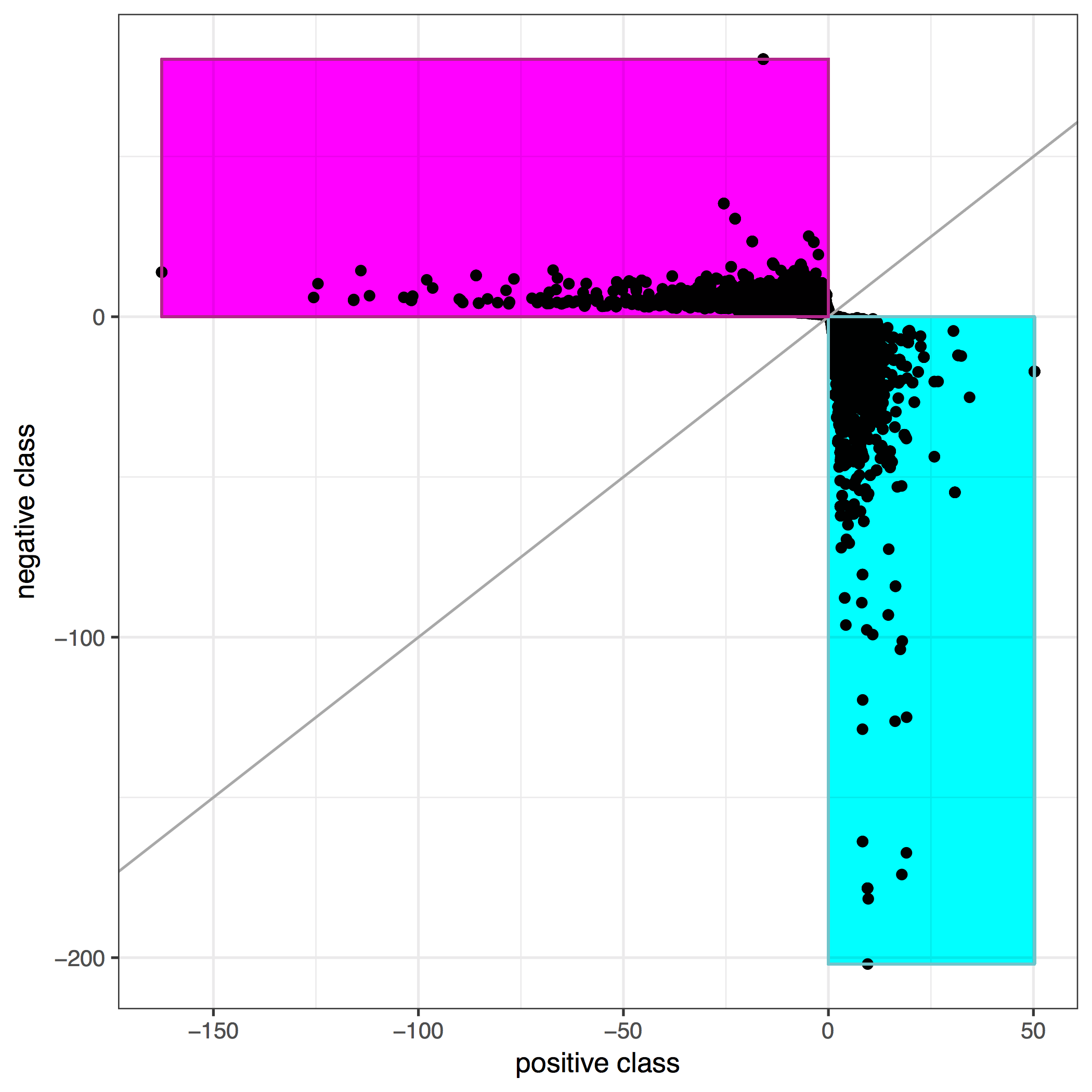


**Figure S8.** GIS-MaxEnt weights for Gene Ontology (GO) term pairs common both to the positive and negative training set: the light blue shading corresponds to GO term pairs contributing to higher score assignment towards the positive class and the violet rectangle highlights the weights that contribute more towards a negative class assignment.

1. Tables:

| GO branch | No. of terms | Levels |
| --- | --- | --- |
| molecular function | 10,040 | 15 |
| cellular component | 3,048 | 12 |
| biological process | 22,259 | 15 |

**Table S1.** Number of GO terms present in each Gene Ontology (v.1.1.2412) branch and the associated (maximum) number of levels.

| Source | Nr of genes covered |
| --- | --- |
| GO-CC | 736 (68.33%) |
| GO-BP | 793 (73.63%) |
| GO-MF | 733 (68.05%) |
| GO | 823 (76.41%) |
| InterPro | 833 (77.34%) |
| GO or InterPro | 841 (78.08%) |
| GO and InterPro | 815 (75.67%) |
| No annotation | 236 (21.92%) |

**Table S2**. Number of proteins in the positive set covered by different annotation sources.

|  | GO-CC | GO-BP | GO-MF | any GO | IP | any GO-IP | GO-and-IP |
| --- | --- | --- | --- | --- | --- | --- | --- |
| positive set (A) | 4,381 | 4,102 | 3,697 | 4,721 | 5,155 | 5,204 | **3,164** |
| negative set (B) | 3,303 | 3,779 | 2,777 | 4,537 | 5,461 | 5,508 | **1,852** |

**Table S3.** Annotation coverage of interactions in the positive (A) and negative (B) set based on the sources used. IP: InterPro, CC: GO cellular compartment, MF: GO molecular function, BP: GO biological process. Each training set contains 9,593 interactions. 3,164 and 1,852 interactions in the positive and negative set respectively had at least one GO and one InterPro annotation.

| Data set | GO-CC | GO-BP | GO-MF | any GO | IP | any GO-IP |
| --- | --- | --- | --- | --- | --- | --- |
| A-500 | 7,905 | 118,191 | 3,679 | 129,775 | 3,462 | 133,237 |
| B-500 | 15,023 | 125,591 | 6,961 | 147,575 | 5,285 | 152,860 |
| A-500 & B-500 | 17,004 | 190,118 | 8,875 | 215,997 | 8,632 | 224,629 |

**Table S4.** Number of unique attributes in each set (positive set (A), negative set (B) and combined training set (A and B)) for each of the different annotation sources. One protein-protein interaction attribute is formed out of two protein annotations, where each annotation belongs to one of the proteins involved.

| $MCC=\frac{TP\times TN-FP\times FN}{\sqrt{(TP+FN)\times(FP+TN)\times(TP+FP)\times(FN+TN)}}$ | $recall=\frac{TP}{TP+FN}$ |
| --- | --- |
| $ACC=\frac{TP+TN}{TP+FN +FP+TN}$ | $precision=\frac{TP}{TP+FP}$ |
| $F1=\frac{2TP}{2TP+FP+FN}$ | $FPR=FP/N$ |
| $FDR=\frac{FP}{FP+TP}$ | $TPR=TP/P$ |

**Table S5**. Evaluation measurements for the generalization performance of classifiers. TP: True Positives, TN: True Negatives, FP: False Positives, FN: False Negatives, MCC: Matthews Correlation Coefficient, ACC: Accuracy, F1: F score, FDR: False Discovery Rate, FPR: False Positive Rate, TPR: True Positive Rate

| Set | ACC | MCC | F1 | Recall | Precision |
| --- | --- | --- | --- | --- | --- |
| GO-BP | 0.758±0.011 | 0.56±0.019 | 0.798±0.008 | 0.938±0.008 | 0.703±0.013 |
| GO-CC | 0.915±0.004 | 0.832±0.008 | 0.916±0.004 | 0.916±0.005 | 0.917±0.006 |
| GO-MF | 0.902±0.004 | 0.806±0.009 | 0.903±0.004 | 0.913±0.005 | 0.895±0.006 |
| GO | 0.93±0.004 | 0.861±0.007 | 0.931±0.004 | 0.944±0.005 | 0.919±0.005 |
| IP | 0.807±0.005 | 0.645±0.01 | 0.772±0.008 | 0.659±0.01 | 0.939±0.005 |
| GO-IP | 0.931±0.004 | 0.863±0.007 | 0.932±0.003 | 0.948±0.005 | 0.918±0.005 |
| GME | 0.949±0.003 | 0.878±0.007 | 0.949±0.003 | 0.941±0.005 | 0.937±0.006 |
| SVM-GO-BP | 0.879±0.005 | 0.759±0.01 | 0.878±0.005 | 0.87±0.006 | 0.888±0.006 |
| SVM-GO-CC | 0.916±0.004 | 0.833±0.008 | 0.918±0.004 | 0.941±0.004 | 0.897±0.005 |
| SVM-GO-MF | 0.882±0.006 | 0.769±0.012 | 0.878±0.007 | 0.851±0.01 | 0.91±0.007 |
| SVM-GO | 0.936±0.003 | 0.873±0.007 | 0.936±0.003 | 0.933±0.005 | 0.939±0.004 |
| SVM-IP | 0.798±0.005 | 0.64±0.009 | 0.751±0.008 | 0.617±0.01 | 0.968±0.004 |
| GO-IP | 0.937±0.003 | 0.875±0.007 | 0.936±0.004 | 0.944±0.004 | 0.929±0.005 |
| MKL | 0.949±0.003 | 0.899±0.007 | 0.949±0.003 | 0.951±0.005 | 0.947±0.004 |

**Table S6.** Performance of the different systems trained on different datasets evaluated using accuracy (ACC), Matthews Correlation Coefficient (MCC), F1, recall and precision as defined in the formulas (Table S5). GIS-MaxEnt trained on the six different training sets: GO-BP, GO-CC, GO-MF, GO, GO-IP, SVM trained on the same six training sets: SVM-GO-BP, SVM-GO-CC, SVM-GO-MF; SVM-GO, SVM-IP, SVM-GO-IP; GIS-MaxEnt Ensemble (GME) and Multiple Kernel Learning (MKL) which were trained on all the data.

| Dataset | GIS-MaxEnt – MCC |  | KL (SVM or MKL) – MCC |
| --- | --- | --- | --- |
| GO-BP | 0.560±0.018 | < | 0.759±0.01 |
| GO-CC | 0.832±0.008 | < | 0.833±0.008 |
| GO-MF | 0.806±0.008 | > | 0.769±0.012 |
| IP | 0.645±0.01 | > | 0.640±0.009 |
| GO-IP | 0.863±0.007 | < | 0.875±0.007 |
| GO-IP ensemble | 0.878±0.007 | < | 0.899±0.007 |

**Table S7.** Comparison summary containing the MCC value for the GIS-MaxEnt and kernel learning based methods. The less than (<) or greater than (>) symbols indicate which model had a better performance.

| GIS-MaxEnt | GO-CC | GO-BP | GO-MF | GO | IP | GO-IP |
| --- | --- | --- | --- | --- | --- | --- |
| GO-CC | 1 | 1.11E-14 | 0.0469488 | 0.01405861 | 1.33E-16 | 0.005923459 |
| GO-BP | NA | 1 | 1.77E-13 | 4.32E-16 | 2.81E-05 | 3.33E-16 |
| GO-MF | NA | NA | 1 | 3.38E-05 | 4.89E-15 | 1.52E-05 |
| GO | NA | NA | NA | 1 | 2.05E-17 | 0.8576522 |
| IP | NA | NA | NA | NA | 1 | 1.34E-17 |
| GO-IP | NA | NA | NA | NA | NA | 1 |

**Table S8**. Two sample Wilcoxon unpaired test *p*-values obtained using Matthews Correlation Coefficients (MCC) for 50 internal partitions of each GIS-MaxEnt trained on different annotation sets.

| SVM | GO-CC | GO-BP | GO-MF | GO | IP | GO-IP |
| --- | --- | --- | --- | --- | --- | --- |
| GO-CC | 1 | 1.80E-07 | 2.18E-05 | 1.30E-03 | 5.21E-17 | 4.35E-04 |
| GO-BP | NA | 1 | 2.51E-01 | 9.79E-14 | 1.19E-12 | 1.13E-13 |
| GO-MF | NA | NA | 1 | 4.67E-12 | 7.27E-13 | 3.36E-12 |
| GO | NA | NA | NA | 1 | 7.72E-18 | 6.89E-01 |
| IP | NA | NA | NA | NA | 1 | 9.14E-18 |
| GO-IP | NA | NA | NA | NA | NA | 1 |

**Table S9.** SVM trained on different annotation sets. *p*-values are from two-sample Wilcoxon unpaired test performed using 50 MCC values from each individual trained model.

|  | SVM | | | | | | |
| --- | --- | --- | --- | --- | --- | --- | --- |
| GIS-MaxEnt |  | GO-CC | GO-BP | GO-MF | GO | IP | GO-IP |
|  | GO-CC | 6.44E-01 | 5.07E-07 | 1.84E-04 | 3.53E-04 | 2.29E-17 | 1.27E-04 |
|  | GO-BP | 6.10E-15 | 9.29E-11 | 3.54E-11 | 1.24E-16 | 5.41E-05 | 9.77E-17 |
|  | GO-MF | 2.59E-02 | 5.72E-04 | 2.01E-02 | 3.21E-07 | 6.57E-16 | 7.87E-08 |
|  | GO | 2.93E-02 | 1.54E-11 | 3.10E-09 | 2.67E-01 | 9.39E-18 | 1.44E-01 |
|  | IP | 2.10E-16 | 3.05E-11 | 9.85E-12 | 1.11E-17 | 8.74E-01 | 1.39E-17 |
|  | GO-IP | 1.55E-02 | 2.70E-12 | 3.14E-10 | 2.88E-01 | 8.82E-18 | 1.53E-01 |

**Table S10**. GIS-MaxEnt versus SVM. Two-sample Wilcoxon unpaired test (*p*-values) performed using 50 Matthews Correlation Coefficient (MCC) values from each individual trained model.

|  | GO-CC | GO-BP | GO-MF | GO | IP | GO-IP |
| --- | --- | --- | --- | --- | --- | --- |
| GME | 5.23E-05 | 1.24E-16 | 4.69E-08 | 5.10E-02 | 1.49E-17 | 9.17E-02 |

**Table S11**. GIS-MaxEnt versus GIS-MaxEnt Ensemble (GME). Two-sample Wilcoxon unpaired test (*p*-values) performed using 50 MCC values from each individual trained model.

| Set | ACC | MCC | F1 | Recall | Precision |
| --- | --- | --- | --- | --- | --- |
| GO-BP | 0.772±0.021 | 0.552±0.012 | 0.789±0.006 | 0.855±0.010 | 0.734±0.007 |
| GO-CC | 0.818±0.008 | 0.637±0.016 | 0.816±0.008 | 0.810±0.011 | 0.825±0.011 |
| GO-MF | 0.666±0.011 | 0.357±0.024 | 0.717±0.009 | 0.847±0.013 | 0.622±0.009 |
| GO | 0.841±0.007 | 0.685±0.013 | 0.846±0.006 | 0.870±0.010 | 0.824±0.009 |

**Table S12**. GIS-MaxEnt performance on an independent 1,379 yeast binary interaction data set evaluated using accuracy (ACC), Matthews Correlation Coefficient (MCC), F1, recall and precision as defined in the formulas (Table S5).

1. Supplementary Notes:

**Supplementary Note 1**. Protein-protein interaction experimental methods

A large fraction of protein-protein interactions recorded in public databases originate from studies using the yeast two-hybrid (Y2H) system (see IRefIndex; (Razic*k et a*l., 2008)). Protein microarrays, in which a subset of (bait) proteins are arrayed on a static substrate and introduced to wider (prey) proteins in order to identify specific direct interactions, have also been used for high-throughput interaction screening (MacBeath and Schreiber, 2000; Kung and Snyder, 2006; Stoevesand*t et a*l., 2009; Tessler and Mitra, 2011; Gupt*a et a*l., 2016). These methods, although very powerful, largely characterise binary interactions (direct interaction between two proteins). Proteins are known to exist in much larger complexes, some with multiple copies of the same protein, with concerted functional activity (Robinso*n et a*l., 2007; Alberts, 1998).

Protein complexes span a wide abundance range, and many are membrane bound and associated with different sub-cellular compartments. Furthermore, proteins interact with varying affinities, many partners bind transiently, and interactions may differ according to the protein isoforms present. These properties present a challenge to reliable high-throughput assays, and this has motivated the application of mass spectrometry (MS) methods to characterise whole complexes on a global scale to measure the presence and abundance of both direct and indirect interacting partners. Such methods include large scale biochemical fractionation of protein complexes (Havugiman*a et a*l., 2012; Kristense*n et a*l., 2012), and affinity purification coupled with mass spectrometry (AP-MS) (Wa*n et a*l., 2015). Additionally, proximity tagging methods such as BioID (Rou*x et a*l., 2012), APEX (Rhe*e et a*l., 2013) and SPPLAT (L*i et a*l., 2014) give information about interaction partners of a bait protein fused with an agent that will biotinylate any proteins that come into close proximity with the bait protein *in vivo*.

Fundamental elements of the macromolecular complexes are conserved across metazoa, at least in terms of the soluble multiprotein complexes detectable by biochemical fractionation coupled with quantitative mass spectrometry as revealed by Wan and colleagues (Wa*n et a*l., 2015).

**Supplementary Note 2**. Application of supervised support vector machines to predict protein-protein interactions and training set design

Previous work has defined the prediction of protein interactions as a binary classification problem requiring two training sets: a positive set containing high confidence interactions and a negative set containing protein pairs likely not to interact. Positive training sets have been designed using several criteria depending on the main aim of the classifier. Miller et al. used manual curation to design a training set for studying integral membrane protein interactions, while others used specific filter criteria, such as PPIs having at least one shared GO term or interacting homologs, on large PPI databases to select a subset as positive training set (Mille*r et a*l., 2005; Patil and Nakamura, 2005; Ben-Hur and Noble, 2005; Qiu and Noble, 2008).

Negative training sets have been generated by random selection of protein pairs with additional criteria, such as requiring the proteins to have different cellular localization or not be listed as interacting in specific databases (Patil and Nakamura, 2005; Ben-Hur and Noble, 2005; Qiu and Noble, 2008).

Subsequent work has focused on combining multiple complementary methods of defining a positive training sets into one comprehensive training set, while specific domain knowledge is ongoing being applied successfully by means of specific training sets and problem setting. A specific problem setting is for example the application of an iterative algorithm to accurately predict specific interaction partners belonging to two protein families, known to share interaction partners such as histidine kinases, response regulators and ATP-binding cassette transporter complexes (Yu and Finley, 2009; Bitbo*l et a*l., 2016).

**Supplementary Note 3**. Maximum Entropy Learning model and implementation package

Maximum Entropy modelling has been successfully applied to natural language processing, where it rewards words with low frequency (high in information content) and penalizes highly frequent words, in the same way root nodes are more often encountered in the GO parents list and are less information rich (Berge*r et a*l., 1996; Pietr*a et a*l., 1997). The information content of an item in an ontology is encoded in its closeness to the root (the closest term to the root is less informative than a leaf term).

We provide a brief explanation of the maximum entropy learning method, and refer the reader to (Berge*r et a*l., 1996) for the details.

We begin with a training sequence
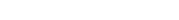
 of
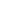
 labelled examples; in the present work the
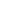
are vectors representing PPIs. Denote by
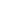
 the empirical distribution for the set
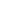


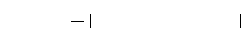


Our aim is to construct a distribution
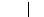
 based on
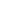
. In doing so we want to ensure that
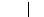
 is consistent in some sense with the underlying behavior of the examples in
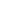
. We therefore introduce the *feature functions*
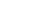
 These will have known expected values


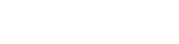


with respect to the empirical distribution. Any model
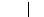
 that we choose will result in corresponding expected values


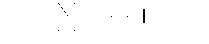


where
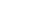
 is the empirical distribution for
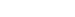
. We would like these to match, so if there are
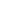
 feature functions then


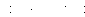
 for
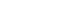
. (1)

The fundamental idea in maximum entropy learning is that we should choose
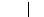
 from the set all possible distributions, such that it respects equation (1) while also maximizing the conditional entropy


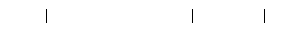


This
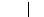
 can be found using standard optimization methods, and the solution can be expressed in the form


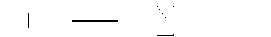


The parameters
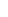
 can be computed using the method of *generalized iterative scaling (GIS).*

GIS-MaxEnt delivers a score between 0 and 1 where 0.5 is assigned to interactions with insufficient information for classification and is used as the decision boundary when predicting binary interactions, while 0 and 1 represent high confidence negative or positive examples respectively. All cases scored with 0.5 were randomly increased or decreased by 0.01 to force a discrete classification for use in subsequent performance evaluation. The model delivers a weight for each annotation, which enables feedback into annotation assignment investigation or training set design.

We used the implementation of the maximum entropy method from version 3.0.0 of the OpenNLP library, available at <https://sourceforge.net/projects/maxent/files/Maxent/3.0.0/>. The original MaxEnt implementation is using as correction constant in the training phase the maximum length of a feature vector. This correction constant was modified to use the median length of the feature vector. This correction constant had resulted in a quicker convergence and improved true positive rate.

**Supplementary Note 4**. Kernel learning

A Support Vector Machine (SVM) seeks to separate the examples of distinct data classes via a hyperplane located at maximal distance to the examples it separates, thereby often referred to as being a large-margin classifier.

Furthermore, exploitation of the ‘kernel trick’ – whereby linear solutions are performed in a non-linear transformation of the original data space – places it among a group of learning algorithms known as kernel methods. The SVM algorithm has become familiar across a wide range of pattern recognition applications, owing primarily to its high performance when properly used (Fernández-Delgad*o et a*l., 2014).

The standalone SVM and the SVM used in the GIS-MaxEnt Ensemble are available in R using the kernlab package (Karatzoglo*u et a*l., 2004), available at https://cran.r-project.org/web/packages/kernlab/index.html.

A natural extension of the SVM is the multiple kernel learning (MKL) algorithm. Rather than specifying a single kernel we learn a linear combination of
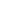
 kernels
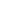


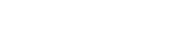


This allows us to choose the individual kernels in the combination to achieve increased flexibility; for example, each
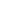
 might depend on only some subset of the elements of
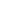
 and
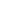
, in which case the values of the corresponding
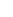
 might be interpreted as indicating the importance of those elements.

Multiple Kernel Learning

In order to apply MKL to the datasets described above, we used an implementation of
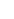
-norm MKL provided by JKernelMachines library (Picar*d et a*l., 2013), which implements the approach of Kloft et al. (Klof*t et a*l., 2011). MKL algorithms typically perform regularization by penalizing choices of the parameter vector
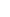
 having a large norm. As a result, use of the
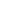
-norm tends to promote sparsity, in the sense that many of the parameters
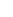
are forced to 0. Alternatively, use of the
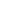
-norm


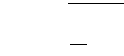


while not promoting sparsity can yield increased accuracy in classification tasks (Klof*t et a*l., 2011).

**Supplementary Note 5.** Model Selection and Estimated Generalization

Algorithmic free-parameters were set using estimated generalisation performance as assessed via a second round of partitioning on each of the 50 training partitions (Figure S7). Generalisation was estimated using stratified 10-fold cross-validation with the Matthews correlation coefficient (MCC) (Matthews, 1975). In the case of the SVM used by the GIS-MaxEnt Ensemble we used class-specific generalisation parameters
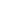
 and
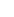
, both optimized over the range of
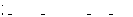
. As the use of class-specific parameters is primarily for the case where the positive and negative classes are unbalanced we reverted to a single parameter
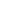
, optimized over the same range, in the case of the standalone SVM and the MKL method.

The final generalization performance was quantified by several measures (Table S5), calculated on the 10% test set and averaged over the 50 partitions. In addition, the Receiver Operating Characteristic (ROC) curve was obtained by plotting average false positive rate (FPR) and true positive rate (TPR) obtained by varying the threshold from 0 to 1 in 100 steps.

**Supplementary Note 6.** Yeast data set

The *S. cerevisiae* 1,379 binary interactions between 624 proteins (Cela*j et a*l., 2017) were used a positive training set. The GO annotation and list of published interactions was retrieved using YeastMine (version: Sept-25-2017) (Balakrishna*n et a*l., 2012). All but one the identifiers maintained at least one GO annotation after the evidence code filtering. YGL242C had only 3 GO annotation terms with evidence code ND (No biological Data available) which were filtered out. This resulted that one out of the 1,379 interactions to have no annotation.

**References**:

Alberts,B. (1998) The cell as a collection of protein machines: preparing the next generation of molecular biologists. *Cell*, **92**, 291–294.

Balakrishnan,R. *et al.* (2012) YeastMine--an integrated data warehouse for Saccharomyces cerevisiae data as a multipurpose tool-kit. *Database*, **2012**, bar062-bar062.

Ben-Hur,A. and Noble,W.S. (2005) Kernel methods for predicting protein-protein interactions. *Bioinformatics*, **21 Suppl 1**, i38-46.

Berger,A.L. *et al.* (1996) A Maximum Entropy Approach to Natural Language Processing. *Comput. Linguist.*, **22**, 39–71.

Bitbol,A.-F. *et al.* (2016) Inferring interaction partners from protein sequences. *arXiv*, **113**, 50732.

Celaj,A. *et al.* (2017) Quantitative analysis of protein interaction network dynamics in yeast. *Mol. Syst. Biol.*, **13**, 934.

Fernández-Delgado,M. *et al.* (2014) Do we need hundreds of classifiers to solve real world xlassification problems? *J. Mach. Learn. Res.*, **15**, 3133–3181.

Gupta,S. *et al.* (2016) An overview of innovations and industrial solutions in Protein Microarray Technology. *Proteomics*, **16**, 1297–308.

Havugimana,P.C. *et al.* (2012) A census of human soluble protein complexes. *Cell*, **150**, 1068–1081.

Karatzoglou,A. *et al.* (2004) kernlab - An S4 Package for Kernel Methods in R. *J. Stat. Softw.*, **11**, 1–20.

Kloft,M. *et al.* (2011) Lp-norm multiple kernel learning. *J. Mach. Learn. Res.*, **12**, 953–997.

Kristensen,A.R. *et al.* (2012) A high-throughput approach for measuring temporal changes in the interactome. *Nat. Methods*, **9**, 907–909.

Kung,L.A. and Snyder,M. (2006) Proteome chips for whole-organism assays. *Nat. Rev. Mol. Cell Biol.*, **7**, 617–622.

Li,X.-W. *et al.* (2014) New insights into the DT40 B cell receptor cluster using a proteomic proximity labeling assay. *J. Biol. Chem.*, **289**, 14434–47.

MacBeath,G. and Schreiber,S.L. (2000) Printing proteins as microarrays for high-throughput function determination. *Science (80-. ).*, **289**, 1760–1763.

Matthews,B.W. (1975) Comparison of the predicted and observed secondary structure of T4 phage lysozyme. *BBA - Protein Struct.*, **405**, 442–451.

Miller,J.P. *et al.* (2005) Large-scale identification of yeast integral membrane protein interactions. *Proc. Natl. Acad. Sci. U. S. A.*, **102**, 12123–12128.

Patil,A. and Nakamura,H. (2005) Filtering high-throughput protein-protein interaction data using a combination of genomic features. *BMC Bioinformatics*, **6**, 100.

Picard,D. *et al.* (2013) JKernelMachines: A simple framework for kernel machines. *J. Mach. Learn. Res.*, **14**, 1417–1421.

Pietra,S. Della *et al.* (1997) Inducing Features of Random Fields. *IEEE Trans. Pattern Anal. Mach. Intell.*, **19**, 380–393.

Qiu,J. and Noble,W.S. (2008) Predicting co-complexed protein pairs from heterogeneous data. *PLoS Comput. Biol.*, **4**, e1000054.

Razick,S. *et al.* (2008) iRefIndex: A consolidated protein interaction database with provenance. *BMC Bioinformatics*, **9**, 405.

Rhee,H.-W. *et al.* (2013) Proteomic mapping of mitochondria in living cells via spatially restricted enzymatic tagging. *Science*, **339**, 1328–31.

Robinson,C. V. *et al.* (2007) The molecular sociology of the cell. *Nature*, **450**, 973–982.

Roux,K.J. *et al.* (2012) A promiscuous biotin ligase fusion protein identifies proximal and interacting proteins in mammalian cells. *J. Cell Biol.*, **196**, 801–10.

Stoevesandt,O. *et al.* (2009) Protein microarrays: high-throughput tools for proteomics. *Expert Rev. Proteomics*, **6**, 145–57.

Tessler,L.A. and Mitra,R.D. (2011) Sensitive single-molecule protein quantification and protein complex detection in a microarray format. *Proteomics*, **11**, 4731–4735.

Wan,C. *et al.* (2015) Panorama of ancient metazoan macromolecular complexes. *Nature*, **525**, 339–44.

Yu,J. and Finley,R.L. (2009) Combining multiple positive training sets to generate confidence scores for protein-protein interactions. *Bioinformatics*, **25**, 105–111.
